# Impact of mirikizumab treatment on fatigue in patients with moderately to severely active Crohn’s disease: results from the phase 3 VIVID-1 study

**DOI:** 10.1093/ecco-jcc/jjaf100

**Published:** 2025-06-10

**Authors:** Miguel Regueiro, Monika Fischer, Peter Bossuyt, Marijana Protic, Kristina Traxler, Guanglei Yu, Huaiyu Zang, Aisha Vadhariya, Tadakazu Hisamatsu, Pascal Juillerat, Alessandro Armuzzi, Javier P Gisbert, Alissa Walsh

**Affiliations:** Department of Gastroenterology, Hepatology, and Nutrition, Cleveland Clinic Foundation, Cleveland, OH, United States; Division of Gastroenterology and Hepatology, Indiana University School of Medicine, Indianapolis, IN, United States; Department of Gastroenterology, Imelda General Hospital, Bonheiden, Belgium; Eli Lilly and Company, Indianapolis, IN, United States; Eli Lilly and Company, Indianapolis, IN, United States; Eli Lilly and Company, Indianapolis, IN, United States; Tigermed-BDM Inc., Somerset, NJ, United States; Eli Lilly and Company, Indianapolis, IN, United States; Department of Gastroenterology and Hepatology, Kyorin University School of Medicine, Mitaka, Japan; Intesto Crohn and Colitis Center, Bern, Switzerland; Department of Visceral Surgery and Medicine, Inselspital Bern University Hospital, University of Bern, Bern, Switzerland; IBD Center IRCCS Humanitas Research Hospital, Rozzano, Milan, Italy; Department of Biomedical Sciences, Humanitas University, Pieve Emanuele, Milan, Italy; Gastroenterology Unit, Hospital Universitario de La Princesa, Instituto de Investigación Sanitaria Princesa (IIS-Princesa), Universidad Autónoma de Madrid (UAM), and Centro de Investigación Biomédica en Red de Enfermedades Hepáticas y Digestivas (CIBEREHD), Madrid, Spain; Translational Gastroenterology Unit, John Radcliffe Hospital, Oxford University Hospitals NHS Foundation Trust and NIHR Biomedical Research Centre, Oxford, United Kingdom

**Keywords:** Crohn’s disease, mirikizumab, fatigue

## Abstract

**Background:**

Fatigue is a debilitating multifactorial symptom experienced by patients with Crohn’s disease (CD). Mirikizumab, an anti-interleukin-23p19 antibody, demonstrated significant efficacy and safety in the patients with moderately to severely active CD. This analysis investigated the impact of mirikizumab on fatigue and the association between changes in clinical, endoscopic, and patient-reported outcomes with improvement in fatigue from baseline in the Phase 3 VIVID-1 study.

**Methods:**

Adult patients with moderately to severely active CD that failed at least 1 biologic agent or conventional therapy were randomized to receive mirikizumab or placebo. Fatigue was assessed via the validated Functional Assessment of Chronic Illness Therapy-Fatigue questionnaire. Fatigue associations with patient-reported outcomes, endoscopic, and clinical measures were assessed via Pearson correlation analysis.

**Results:**

At Week 12, 43% and 33%, and at Week 52, 46% and 36% of mirikizumab-treated patients achieved ≥ 6 and ≥ 9 fatigue score improvements vs placebo (Week 12, 31%, 22%; Week 52, 20%, 16%), respectively. Baseline fatigue scores were strongly associated with depressive symptoms and moderately associated with quality of life (QoL) at baseline. Improvements in fatigue at Weeks 12 and 52 were strongly associated with QoL and patient-reported outcomes and weakly with objective markers of inflammation and disease activity.

**Conclusions:**

Mirikizumab-treated patients with CD achieved higher rates of clinically meaningful improvement in fatigue vs placebo at Weeks 12 and 52, which correlated with improvement in clinical and patient-reported outcomes. Baseline fatigue severity was strongly associated with depressive symptoms in VIVID-1 (NCT03926130).

## 1. Introduction

Crohn’s disease (CD) is a debilitating, chronic, and progressive inflammatory bowel disease (IBD) with symptoms including diarrhea, abdominal pain (AP), and fatigue.^1^

Fatigue has been described as the persistent sense of tiredness, weakness, or exhaustion that impacts physical and mental work but is not relieved by rest.^2^ Up to 86% of patients with active CD report fatigue, and up to 54% of patients with CD report fatigue while in remission.^3,4^ Fatigue is a multidimensional symptom which may include the subjective feelings of weakness, objective measures of decreased activity, difficulty concentrating, and decreases in motivation and mood.^5^ Additionally, it has been reported that up to 61% of patients indicate that fatigue significantly impacts their quality of life (QoL).^6^ The primary causes of fatigue are incompletely understood, and it remains challenging to explain fatigue persistence despite clinical and endoscopic improvement.

In the Phase 3 VIVID-1 study, mirikizumab, a humanized IgG4 monoclonal antibody, achieved and maintained symptomatic improvement, clinical remission, and endoscopic remission in CD.^7^ Here, we investigate the effects of mirikizumab on fatigue, and the relationship of fatigue with clinical, endoscopic, and patient-reported outcomes endpoints at baseline, Week 12, and Week 52.

## 2. Methods

### 2.1. Study design

VIVID-1 (NCT03926130) is a Phase 3, multicenter, randomized, double-blind, double-dummy, parallel-group, placebo- and active-controlled treat-through study.^7^ The analysis in this manuscript includes the mirikizumab and placebo arms only. The VIVID-1 protocol was approved by local ethical review boards and conducted according to the International Conference on Harmonisation Good Clinical Practice guidelines and the Declaration of Helsinki.

### 2.2. Patients

The VIVID-1 study enrolled adult patients with moderately to severely active CD defined by average daily stool frequency (SF) ≥ 4 and/or average daily AP score  ≥ 2 at baseline and endoscopic evidence of mucosal inflammation by the Simple Endoscopic Score for Crohn’s disease (SES-CD score) ≥ 7 (or ≥ 4 for patients with isolated ileal disease) and also demonstrated intolerance, inadequate response, or loss of response to conventional, or to biologic therapies. Eligibility requirements are fully described elsewhere.^7^

### 2.3. Assessment

Fatigue was measured using the Functional Assessment of Chronic Illness Therapy (FACIT)-Fatigue, which is a 13-item scale measuring self-reported fatigue and its impact on individuals with chronic illnesses. FACIT-Fatigue total score ranges from 0 to 52 where higher scores indicate less severe fatigue.^8^ It has been previously validated in patients with IBD,^9^ and specifically CD.^10^ The FACIT-Fatigue was completed during study visits at Weeks 0, 12, and 52. FACIT-Fatigue total scores in VIVID-1 were not calculated if 8 or more items were missing. Psychometric analysis of data from the VIVID-1^10^ study showed that a 6–9-point improvement in FACIT-Fatigue score represents a clinically meaningful improvement (CMI) in the adults with moderately to severely active CD which is consistent with other studies.^11,12^

### 2.4. Outcomes

#### 2.4.1. Efficacy in improvement of fatigue

In this treat-through trial, placebo non-responders were switched to mirikizumab at Week 12, therefore composite endpoints (clinical response by Patient-Reported Outcome [PRO] at Week 12 and FACIT-Fatigue CMI at Week 52) were used for fair comparison between mirikizumab and placebo. Clinical response by PRO was defined as ≥ 30% decrease in very soft or liquid SF or AP and neither worse than baseline.

The evaluated endpoints for mirikizumab vs placebo included: mean change from baseline of FACIT-Fatigue scores at Week 12 and Week 52, and patients who achieved the composite endpoint of Week 12 clinical response by PRO and ≥ 6 and ≥ 9-point improvement in FACIT-Fatigue from baseline at Week 52.

#### 2.4.2. Factors associated with fatigue at baseline

To understand factors potentially impacting fatigue severity in patients with CD, associations of baseline characteristics with baseline FACIT-Fatigue were assessed in patients randomized to either mirikizumab or placebo. These baseline characteristics included baseline scores of clinical and endoscopic disease severity, inflammatory biomarkers, previous disease history (duration of disease, previous surgeries, corticosteroid use, biologic experience), and QoL measurements. These factors were selected according to the clinical understanding of the potential associations and previously published data.^11,13–15^ A complete list of measures and their description is presented in Table S1.

#### 2.4.3. Association of fatigue improvement with other endpoints at week 12 and week 52

Based on previous studies that revealed fatigue may persist CD remission, the association of FACIT-Fatigue CMI at Week 12 and Week 52 with improvement of clinical, endoscopic, biomarkers and QoL parameters among mirikizumab-treated patients was evaluated. We analyzed the following:

1. Association of change in FACIT-Fatigue score at Week 12 and Week 52 with change from baseline in the following continuous endpoints: AP score, SF score, Urgency Numeric Rating Scale (NRS), log-transformed fecal calprotectin (FCP), log-transformed C-reactive protein (CRP), Inflammatory Bowel Disease Questionnaire (IBDQ) domain scores, 36-Item Short Form Survey (SF-36) physical component summary (PCS) and mental component summary (MCS) scores, Crohn’s Disease Activity Index (CDAI) total score, SES-CD total score, hematocrit (HCT), hemoglobin, Robarts Histopathology Index, and the 16-item Quick Inventory of Depressive Symptomatology-Self-Report (QIDS-SR16).
2a. Agreements in achieving FACIT-Fatigue CMI (with baseline FACIT-F score ≤ 46 for ≥ 6 improvement and with baseline FACIT-F score ≤ 43 for ≥ 9 improvement) at Week 12 at the following endpoints: IBDQ response, clinical remission by PRO, clinical remission by CDAI, and endoscopic response.
2b. Agreements in achieving FACIT-Fatigue CMI at Week 52 at the following endpoints: IBDQ remission, clinical remission by CDAI, corticosteroid-free CDAI clinical remission, endoscopic response, and endoscopic remission.
3a. Ranking predictor importance in achieving CMI in FACIT-Fatigue at Week 12 with the following variables: baseline QIDS-SR16, baseline CDAI total score, baseline log-transformed CRP, baseline immunomodulator use, number of surgical bowel resection, duration of CD, disease location, baseline log-transformed FCP, baseline Urgency NRS, age at diagnosis, SES-CD total score, baseline corticosteroid use, presence of perianal disease, and baseline HCT.
3b. Ranking predictor importance in achieving CMI in FACIT-Fatigue at Week 52 with the following variables: baseline QIDS-SR16, baseline CDAI total score, baseline log-transformed CRP, baseline immunomodulator use, number of surgical bowel resection, duration of CD, disease location, baseline log-transformed FCP, baseline Urgency NRS, age at diagnosis, SES-CD total score, baseline corticosteroid use, presence of perianal disease, baseline HCT, Week 12 change from baseline in AP average, Week 12 endoscopic response, Week 12 change from baseline Urgency NRS, Week 12 clinical remission by CDAI, Week 12 change from baseline in SF average, Week 12 log-transformed CRP change from baseline, Week 12 clinical response by PRO, and Week 12 log-transformed FCP change from baseline.

### 2.5. Statistical analysis

The comparison in change from baseline in FACIT-Fatigue score of patients treated with mirikizumab vs placebo at Week 12 and 52 was performed by analysis of covariance model with least squares mean (LSM) and *P*-value reported.

The comparison in proportions of patients on mirikizumab vs placebo who achieved FACIT-Fatigue ≥ 6-point and ≥ 9-point increase at Week 12 and composite endpoints of Week 12 clinical response by PRO and a FACIT-Fatigue ≥ 6 and ≥ 9-point increase at Week 52 was performed by Cochran-Mantel-Haenszel test. Additional placebo-anchored matching-adjusted indirect comparisons (MAICs) were used to compare the FACIT-Fatigue CMI ≥ 9 at Week 12 between mirikizumab and risankizumab (600 mg or 1200 mg) with study-level matching.

The correlation between fatigue severity and other disease characteristics at baseline was assessed using a random forest regression model. This random forest model ranks the correlation strength of each variable with fatigue at baseline based on feature importance, measured by the percentage increase in mean squared error (%IncMSE), with a higher %IncMSE value indicating greater relative importance of the variable.

The agreement of achievement of FACIT-Fatigue ≥ 6-point and ≥ 9-point increase with binary outcomes was evaluated by Cohen’s Kappa coefficient (κ) at Weeks 12 and 52.

The association of change from baseline in FACIT-Fatigue score with other continuous outcomes was evaluated by Pearson correlation coefficient (r) at Weeks 12 and 52. The strength of correlation based on the absolute value of Pearson correlation coefficient is defined as follows: 0.10 to 0.30 weak; > 0.30 to 0.50 moderate; > 0.50 to 1 strong.^16^

Multivariable logistic regression analyses were performed to evaluate the association of baseline or early outcomes on achieving FACIT-F improvement of ≥ 6 and ≥ 9 from baseline at Weeks 12 or 52. The candidate model predictors were pre-specified based on clinical relevance, including baseline characteristics and improvement of disease activity (endoscopy and biomarkers) and QoL endpoints. The importance of individual predictors on FACIT-Fatigue improvement responder was determined using Wald chi-square statistic penalized by the predictor degrees of freedom. The effects of predictors were reported by odds ratios (ORs), and the corresponding CIs were calculated using Wald statistics. The ORs were based on interquartile ranges for continuous variables. Missing post-baseline data are handled by non-responder imputation for binary endpoints and modified baseline observation carried forward for continuous endpoints.

All statistical analyses were conducted using SAS version 9.4 (SAS Institute Inc.) or R version 4.3.2 (R Foundation for Statistical Computing), including the rms package (version 6.7.1) for regression modeling, and randomForest package (version 4.7.1.1) for the random forest method. All *P*-values were 2-sided, and < .05 were considered statistically significant.

## 3. Results

At baseline, the mean (SD) FACIT-Fatigue score was 32.3 (11.1) and 31.5 (11.6) for patients randomized to placebo and mirikizumab, respectively. Demographics and characteristics were similar across treatment groups (Table 1).

**Table 1.** Baseline characteristics and demographics.

	MIRI	PBO
	*N* = 579	*N* = 199
**Age (years), mean (SD)**	36.0 (13.2)	36.3 (12.7)
**Male, *n* (%)**	332 (57.3)	118 (59.3)
**Weight (kg), mean (SD)**	68.0 (18.3)	69.6 (19.0)
**BMI, mean (SD)**	23.2 (5.4)	23.8 (5.8)
**Race, *n* (%)**		
White	408 (71.5)	144 (74.6)
Black or African American	10 (1.8)	5 (2.6)
Asian	148 (25.9)	42 (21.8)
American Indian or Alaska Native	2 (0.4)	2 (1.0)
Multiple	3 (0.5)	0 (0.0)
**Geographic region, *n* (%)**		
Europe and the Rest of the World	319 (55.1)	117 (58.8)
North America	77 (13.3)	27 (13.6)
Central American or South America	30 (5.2)	9 (4.5)
Asia	153 (26.4)	46 (23.1)
**Duration of Crohn’s disease (years), mean (SD)**	7.4 (8.2)	7.8 (7.4)
**Disease location, *n* (%)**		
Ileum only	65 (11.2)	19 (9.5)
Colon only	225 (38.9)	77 (38.7)
Ileum and colon	289 (49.9)	103 (51.8)
**CDAI, mean (SD)**	323.1 (85.8)	318.9 (86.2)
SF daily average, mean (SD)	5.7 (3.0)	5.8 (3.2)
AP daily average, mean (SD)	2.1 (0.6)	2.1 (0.6)
**SES-CD total score, mean (SD)**	13.5 (6.6)	13.1 (6.0)
**CRP (mg/L), median (Q1, Q3)**	8.5 (2.9, 25.0)	7.6 (2.9, 18.8)
**Fecal calprotectin (mg/kg), median (Q1, Q3)**	1315.0 (444.0, 2676.0)	1161.0 (324.0, 2170.0)
**Corticosteroid use, *n* (%)**	177 (30.6)	58 (29.1)
**Immunomodulator use, *n* (%)**	146 (25.2)	58 (29.1)
**Prior biologic failure, *n* (%)**	281 (48.5)	97 (48.7)
**Prior anti-TNF failure, *n* (%)**	265 (45.8)	89 (44.7)
**Prior anti-integrin failure, *n* (%)**	68 (11.7)	24 (12.1)
**Number of failed biologics, *n* (%)**		
None	298 (51.5)	102 (51.3)
1	175 (30.2)	66 (33.2)
≥ 2	106 (18.3)	31 (15.6)
**Urgency NRS, mean (SD)**	6.6 (2.1)	6.6 (2.1)
**IBDQ Total Score, mean (SD)**	127.4 (33.2)	131.2 (32.4)
**QIDS-SR16, mean (SD)**	6.8 (4.8)	6.5 (4.1)
**FACIT-Fatigue, mean (SD)**	31.5 (11.6)	32.3 (11.1)
**Baseline fistulae category, *n* (%)**		
None	541 (93.4)	181 (91.0)
≥ 1	38 (6.6)	18 (9.0)

Abbreviations: AP, abdominal pain; APS, abdominal pain score; BMI, body mass index; CD, Crohn’s disease; CDAI, Crohn’s Disease Activity Index; CRP, C-reactive protein; FACIT, Functional Assessment of Chronic Illness Therapy; MIRI, mirikizumab; *N*, number of patients in the analysis population; *n*, number of patients in the specified category; NRS, Numeric Rating Scale; PBO, placebo; Q, quartile; SES-CD, Simple Endoscopic Score for Crohn’s disease; SF, stool frequency; TNF, tumor necrosis factor; QIDS-SR16, 16-item quick inventory of depressive symptomatology-self-report.

### 3.1. Efficacy

**Figure 1.**
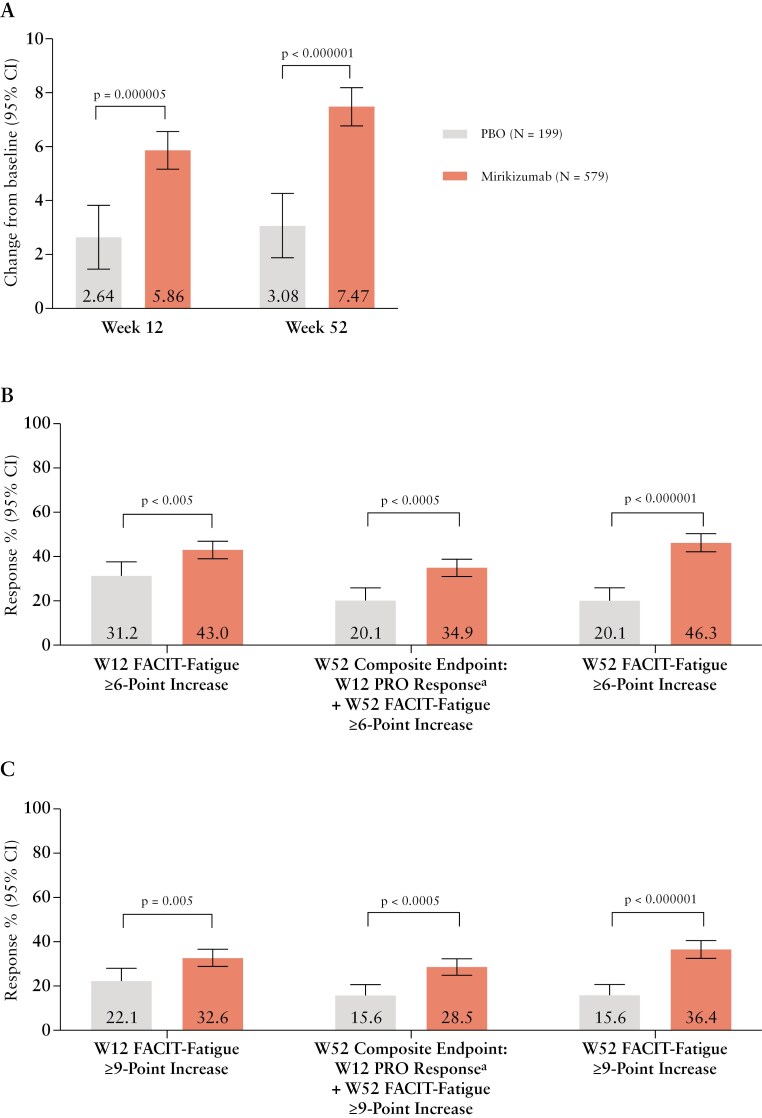
FACIT-Fatigue score assessment in patients randomized to mirikizumab and placebo. (A) Data are LSM (95% CI), and comparisons were performed using ANCOVA with mBOCF. For patients in the placebo group who switched to mirikizumab at Week 12, baseline values were carried forward to derive the change from baseline at Week 52. (B-C) Data are (%) unless otherwise stated. Comparisons were performed using Cochran-Mantel-Haenszel test. ^a^ ≥ 30% decrease in SF or AP, and neither worse than baseline. ANCOVA, analysis of covariance; AP, abdominal pain; FACIT, Functional Assessment of Chronic Illness Therapy; mBOCF, modified baseline observation carried forward; PRO, Patient-Reported Outcome; SF, stool frequency.

At Week 12, mirikizumab-treated patients showed statistically significant improvement adjusted for multiplicity in LSM change from baseline for FACIT-Fatigue (LSM difference = 3.22; *P* < .001) compared to placebo. At Week 52 mirikizumab-treated patients also had greater LSM change from baseline for FACIT-Fatigue (LSM difference = 4.39; *P* < .001) when compared to the placebo group (Figure 1A).

At Week 12, 43.0% of mirikizumab-treated patients and 31.2% of patients receiving placebo achieved fatigue improvement measured by a FACIT-Fatigue ≥ 6-point increase (*P* < .005). At Week 52, 34.9% of mirikizumab-treated patients and 20.1% of patients receiving placebo achieved the composite endpoint of Week 12 PRO response and a FACIT-Fatigue ≥ 6-point increase (*P* < .0005), while 46.3% of mirikizumab-treated patients and 20.1% of patients receiving placebo achieved FACIT-Fatigue ≥ 6-point increase independently of Week 12 PRO response (Figure 1B).

At Week 12, 32.6% of mirikizumab-treated patients and 22.1% of patients receiving placebo achieved FACIT-Fatigue ≥ 9-point increase (*P* = .005). At Week 52, 28.5% of mirikizumab-treated patients achieved composite endpoint of Week 12 PRO response and FACIT-Fatigue ≥ 9-point increase (*P* < .0005), while 36.4% of mirikizumab-treated patients and 15.6% of patients receiving placebo achieved FACIT-Fatigue ≥ 9-point increase independently of Week 12 PRO response (Figure 1C).

In unadjusted indirect comparison or MAIC analysis, there was no statistically significant difference between mirikizumab and risankizumab (600 mg or 1200 mg) in achieving FACIT-Fatigue CMI at Week 12 (Tables S2 and S3).

### 3.2. Factors associated with fatigue at baseline

**Figure 2.**
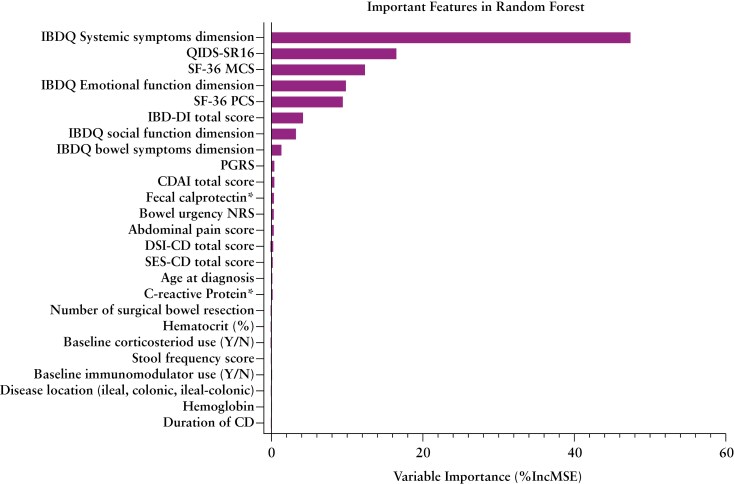
Baseline FACIT-Fatigue scores association analysis for patients randomized to mirikizumab and placebo. Random forest regression model was applied to rank variables by importance associated with FACIT-F scores at baseline. Variable importance was measured by the percentage increase in mean squared error (ie, %IncMSE) after permuting a specific predictor in the random forest model; higher percentages indicate greater importance. *Data for fecal calprotectin and C-reactive protein are log-transformed. CDAI=Crohn’s Disease Activity Index; CRP=C-reactive protein; DSI-CD, Disease Severity Index-Crohn’s Disease; IBDQ, Inflammatory Bowel Disease Questionnaire; IBD-DI, Inflammatory Bowel Disease-Disability Index; Miri, mirikizumab; NRS, numeric rating scale; PGRS, Patient Global Rating of Severity; SES-CD, Simple Endoscopic Score for Crohn’s Disease.

Inflammatory Bowel Disease Questionnaire systemic symptoms domain was the top factor based on the random forest regression model (%IncMSE = 47.36) with a strong positive correlation (r = 0.784) with baseline fatigue (Figure 2; Table S4). Strong associations were observed between baseline fatigue and depressive symptoms using the QIDS-SR16 (%IncMSE = 16.52; r = −0.689), the MCS of SF-36 (%IncMSE = 12.37; r = 0.654), and IBDQ emotional function domain score (%IncMSE = 9.87; r = 0.686). Moderate associations were observed between baseline fatigue scores and QoL measurements, while objective measurements of inflammation, disease activity, prior therapeutic use, and other disease characteristics had weak correlations with fatigue severity at baseline (Figure 2; Table S4).

### 3.3. Association of fatigue improvement with other endpoints at week 12 and week 52

**Figure 3.**
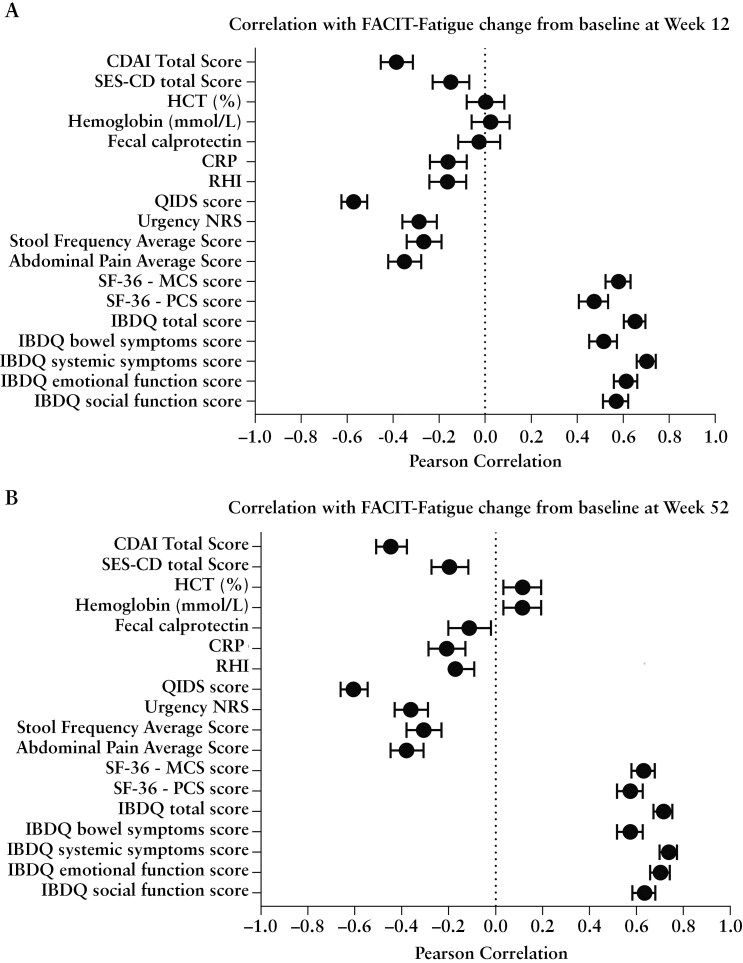
Correlation of change from baseline in FACIT-Fatigue with change from baseline in clinical and quality of life outcomes at Week 12 (A) and Week 52 (B). The absolute value of Pearson correlation is 0.10-0.30 as weak, > 0.30-0.50 as moderate, and > 0.50 to 1 as strong. Pearson correlation is plotted with 95% confidence interval limits. NRS, numeric rating scale; CDAI, Crohn’s Disease Activity Index; CRP, C-reactive protein; Hb, Hemoglobin; HCT, Hematocrit; IBDQ, Inflammatory Bowel Disease Questionnaire; MCS, mental component summary score; PCS, physical component summary score; QIDS, Quick Inventory of Depressive Symptomatology; RHI, Robarts Histopathology Index(Total Colon); SES-CD, Simple Endoscopic Score for Crohn’s Disease.

At Week 12, change from baseline in FACIT-Fatigue showed strong correlations with change from baseline in IBDQ systemic symptoms score (r = 0.702), IBDQ total score (r = 0.652), IBDQ emotional function (r = 0.613) and social function (r = 0.570), IBDQ bowel symptoms (r = 0.515), SF-36 MCS (r = 0.580), and QIDS-SR16 (r = -0.572). For IBDQ total and domain scores and SF-36 MCS, higher scores indicate better QoL and correlate positively with improved FACIT-Fatigue. For QIDS-SR16, higher scores indicate greater symptom severity and correlate negatively with improved FACIT-Fatigue. Moderate to weak correlations were observed between change from baseline in objective measurements of inflammation and disease activity (Figure 3A; Table S5).

At Week 52, similar findings were observed with strong correlations of change from baseline in FACIT-Fatigue with change from baseline in IBDQ systemic symptoms (r = 0.738), IBDQ total score (r = 0.716), IBDQ emotional function (r = 0.703) and social function (r = 0.635), SF-36 MCS (r = 0.631), QIDS-SR16 (r = -0.606), IBDQ bowel symptoms (r = 0.574), and SF-36 PCS (r = 0.574). Moderate to weak correlations were observed between change from baseline in objective measurements of inflammation and disease activity (Figure 3B; Table S5).

### 3.4. Agreement of CMI FACIT-Fatigue at week 12 and week 52 with clinical and QoL outcomes

Inflammatory Bowel Disease Questionnaire response (κ = 0.340, 95% CI, 0.269-0.410) and clinical outcomes had fair agreement with achieving FACIT-Fatigue ≥ 6 improvement at Week 12, while endoscopic response showed weak to no agreement (Table 2). Patients with FACIT-Fatigue ≥ 9 improvement at Week 12 had similar results as ≥ 6-point improvement (Table 2).

**Table 2.** Agreement of≥6 and≥9-point improvement in FACIT-fatigue from baseline at Weeks 12 and 52 with other endpoints.

Variables	Kappa coefficient (95% CI)	
Week 12		
	Achieved ≥ 6-point improvement in FACIT-Fatigue from baseline	Achieved ≥ 9-point improvement in FACIT-Fatigue from baseline
IBDQ response	0.340 (0.269-0.410)	0.289 (0.222-0.357)
Clinical response by PRO	0.213 (0.139-0.287)	0.256 (0.185-0.327)
Clinical remission by CDAI	0.205 (0.124-0.287)	0.288 (0.139-0.317)
Endoscopic response	0.068 (-0.014-0.149)	0.025 (-0.064-0.114)
Week 52		
	Achieved ≥ 6-point improvement in FACIT-Fatigue from baseline	Achieved ≥ 9 point improvement in FACIT-Fatigue from baseline
IBDQ remission	0.406 (0.329-0.484)	0.387 (0.306-0.467)
Clinical remission by CDAI	0.324 (0.243-0.404)	0.298 (0.214-0.383)
Corticosteroid-free CDAI clinical remission	0.321 (0.240-0.401)	0.290 (0.204-0.375)
Clinical remission by PRO	0.309 (0.227-0.390)	0.303 (0.219-0.387)
Endoscopic response	0.181 (0.097-0.265)	0.174 (0.086-0.263)
Endoscopic remission	0.089 (0.013-0.164)	0.111 (0.026-0.195)

*N* = 527 the population only includes patients with a baseline FACIT-F ≤ 46 and randomized to mirikizumab.

*N* = 477 the population includes patients with baseline FACIT-Fatigue ≤ 43 randomized to mirikizumab.

Non-response imputation for imputing missing binary outcomes and modified baseline observation carried forward for imputing missing continuous outcomes. Cohen suggested the Kappa result be interpreted as follows: values ≤ 0 as indicating no agreement and 0.01–0.20 as none to slight, 0.21–0.40 as fair, 0.41– 0.60 as moderate, 0.61–0.80 as substantial, and 0.81–1.00 as almost perfect agreement.^17^.

Abbreviations: CDAI, Crohn’s disease activity index; FACIT, Functional Assessment of Chronic Illness Therapy; IBDQ, Inflammatory Bowel Disease Questionnaire; PRO, patient-reported outcomes.

In patients with FACIT-Fatigue ≥ 6 improvement at Week 52, IBDQ remission (κ = 0.406, 95% CI, 0.329-0.484) showed moderate agreement while clinical outcomes showed fair agreement, and endoscopic outcomes showed weak to no agreement (Table 2). Patients with FACIT-Fatigue improvement of ≥ 9 at Week 52 had fair agreement with IBDQ remission (κ = 0.387, 95% CI, 0.306-0.467) and clinical outcomes, while endoscopic outcomes showed weak to no agreement (Table 2).

### 3.5. Predictors of fatigue improvement at week 12 and week 52

**Figure 4.**
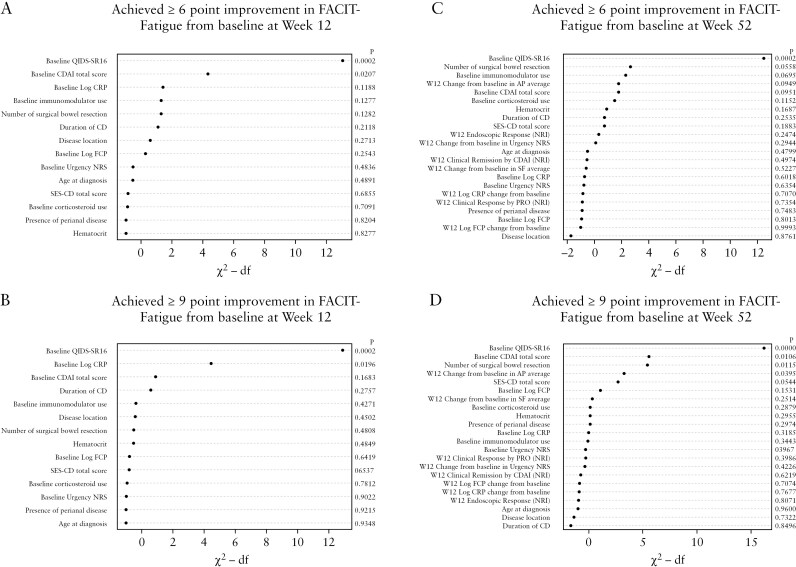
Ranking predictor importance with FACIT-Fatigue scores at Weeks 12 (A, B) and 52 (C, D). Ranking of predictor importance, measured by Wald chi-square value minus the degree of freedom (df) of the predictor, based on multivariable logistic regression. X-axis is represented as “Chi-squared minus degrees of freedom,” which indicates the strength of the predictor variable’s impact on the outcome and its explanatory power in the model. A larger value of Wald chi-square minus df indicates a higher importance of the predictor, meaning it provides more explanatory power to the model. Corresponding *P*-values < .05 indicate that the predictor variable contributes significantly to model.

In multivariable regression modeling, baseline QIDS-SR16 (*P* < .001) and baseline CDAI total score (*P* = .021) were determined to be important predictors for the early (Week 12) 6-point fatigue improvement (Figure 4A). For early (Week 12) FACIT-Fatigue improvement ≥ 9, baseline QIDS-SR16 (*P* < .001) and baseline log-transformed CRP value (*P* = .020) were important predictors (Figure 4B).

At Week 52, for FACIT-Fatigue improvement of ≥ 6, baseline QIDS-SR16 (*P* < .001) was the only important factor among candidate variables included in the model. For FACIT-Fatigue improvement of ≥ 9 at Week 52, important predictors included baseline QIDS-SR16 (< 0.001), baseline CDAI total score (*P* = .011), the number of surgical bowel resections (*P* = .012), and change from baseline in AP at Week 12 (*P* = .040) (Figure 4C and D).

Mirikizumab-treated patients with a higher baseline QIDS-SR16 score had significantly greater odds of achieving the FACIT-Fatigue CMI at both Week 12 (≥ 6: OR = 1.71, 95% CI, 1.29-2.26; ≥ 9: OR = 1.91, 95% CI, 1.36-2.69) and Week 52 (≥ 6: OR = 1.72, 95% CI, 1.29-2.30; ≥ 9: OR = 2.19, 95% CI, 1.51-3.17), and mirikizumab-treated patients with a higher baseline log-transformed CRP value had significantly greater odds of achieving FACIT-Fatigue CMI ≥ 9 at only Week 12 (OR = 1.62, 95% CI, 1.08-2.42). A higher baseline CDAI total score was associated with greater odds of achieving FACIT-Fatigue CMI ≥ 6 at Week 12 (OR = 1.53, 95% CI, 1.07-2.19) and CMI ≥ 9 at Week 52 (OR = 1.83, 95% CI, 1.15-2.92). Mirikizumab-treated patients with a higher number of surgical bowel resections (OR = 0.03, 95% CI, 0.002-0.45) or increase (ie, worsening) of AP from baseline at Week 12 (OR = 0.53, 95% CI, 0.29-0.97) had lower odds of achieving the FACIT-Fatigue CMI ≥ 9 at Week 52 (Figure S1).

## 4. Discussion

In this Phase 3 study, mirikizumab treatment in patients with moderately to severely active CD resulted in consistent and meaningful FACIT-Fatigue score improvements from baseline and higher percentages of ≥6 and ≥9-points improvement at Week 12 and Week 52 compared to patients receiving placebo. At Week 12, 43% of patients on mirikizumab achieved an improvement of 6 or more points in the FACIT-Fatigue scale, while at Week 52, 46% achieved a 6-point or greater improvement independently of PRO response at Week 12. Similar results were observed for ≥9-point improvement in FACIT-Fatigue. Both risankizumab and upadacitinib disclosed similar results in improving FACIT-Fatigue,^11,15^ supporting the effectiveness of newer treatment strategies in managing CD symptoms and improving patients’ QoL. However, differences in clinical trial design and baseline disease characteristics prevent direct comparisons between treatment effects on fatigue among those molecules.

The pathophysiology of fatigue is not well understood.^18^ One hypothesis suggests that fatigue is mediated by inflammatory cytokines,^19^ primarily through the brain-gut axis. The altered gut microbiome in IBD may impact the gut-brain axis, through increased permeability of the gut and consecutively of the blood-brain barrier.^20^ Previous research has also demonstrated that the Th17 intestinal activation plays an important role in signaling astrocytes and meningeal cells and activating central nervous system (CNS)-associated immune response.^21^ In addition, there is a growing body of evidence that Th17 inflammatory axis may contribute to the pathophysiology of depression.^22,23^ These data suggest that mirikizumab may provide benefits to improve non-intestinal symptoms given its impact on both mucosal and systemic inflammation.^7^ Considering the multidimensional nature of fatigue, we explored the association with a wide variety of baseline disease characteristics and disease severity along with previous and current treatment.

Similar to the observation in the Phase 2 mirikizumab program, no associations with objective markers of inflammation were found at baseline.^13^ Different statistical analyses (Figure 2, Table S4) showed that the strongest association was with systemic symptoms reported via IBDQ, which is aligned with the medical constructs included in this domain such as fatigue, energy level, trouble with sleeping, general unwellness, and weight loss (Table S6). Similarly, QIDS-SR16 was strongly associated with fatigue which was expected given the overlap of constructs such as energy levels, appetite, and general interest in people/activities with the FACIT-Fatigue measure.^24^ In alignment with other studies,^25–27^ we found that baseline fatigue severity was strongly associated with depressive symptoms, and mental and emotional status at baseline. This is also consistent with a study^28^ which found that depression primarily influenced fatigue in their IBD study population.

Due to the similarity of the FACIT-Fatigue items with the IBDQ systemic domain items, this strong correlation was expected. At both Weeks 12 and 52, only the improvement of IBDQ score and clinical outcomes was in fair to moderate agreement with fatigue amelioration.

At every time point assessed, fatigue improvement was associated with depressive symptomatology, and mental and emotional status. One interpretation of SERENITY^10^ and VIVID-1 results could be that the effect of mirikizumab on fatigue improvement was achieved through the improvement of psychological well-being independently of alterations in the inflammatory pathway. These results may imply that early psychological support within a multidisciplinary treatment approach may be beneficial for patients with CD, specifically among those in remission with refractory fatigue.

When looking at the predictive factors for early (Week 12) or late (Week 52) fatigue improvement among mirikizumab-treated patients, we observed that those with higher baseline QIDS-SR16 scores had significantly greater odds of achieving CMI in FACIT-Fatigue at both Week 12 and Week 52 (Figure 4). This finding seems to be counterintuitive and could also reflect the potential for a greater magnitude of change with higher baseline severity.

Limitations of the present study include that this is a global clinical trial and most patients were White or Asian which may limit the generalizability of the results. FACIT-Fatigue is a patient-reported outcome measuring fatigue over the last 7 days, where recall bias may exist. Reporting overall improvement in scores or domain scores rather than the fatigue-specific questions increases the difficulty of drawing conclusions on the resolution of fatigue symptoms in each patient-based correlation and association analysis. Unmeasured variables such as sleep, anxiety, depression, and the use of non-pharmacological treatments of fatigue (such as psychiatric therapy) have been reported to impact fatigue in IBD patients but were not controlled for in the analysis.^20,29^

## 5. Conclusions

Treatment with mirikizumab resulted in CMI in fatigue measured by FACIT-Fatigue scores compared to placebo in patients with moderately to severely active CD. Fatigue severity at baseline was associated with QoL. Additionally, improvement in fatigue correlated with improvement in PRO and clinical outcomes at both Weeks 12 and 52. Overall, these results feature the improvement in fatigue severity in patients treated with mirikizumab and highlight valuable information for healthcare providers on fatigue management among patients with CD.

## Supplementary Material

jjaf100_suppl_Supplementary_Tables_S1-S6_Figure_S1

## Data Availability

Lilly provides access to all individual participant data collected during the trial, after anonymization, with the exception of pharmacokinetic or genetic data. Data are available to request 6 months after the indication studied has been approved in the United States and EU and after primary publication acceptance, whichever is later. No expiration date of data requests is currently set once data are made available. Access is provided after a proposal has been approved by an independent review committee identified for this purpose and after receipt of a signed data-sharing agreement. Data and documents, including the study protocol, statistical analysis plan, clinical study report, blank or annotated case report forms, will be provided in a secure data-sharing environment. For details on submitting a request, see the instructions provided at www.vivli.org.
