# Supplementary material for: Impact of mirikizumab treatment on fatigue in patients with moderately to severely active Crohn’s disease: results from the phase 3 VIVID-1 study
Source: J Crohns Colitis. 2025 Jun 10;19(7):jjaf100. doi: 10.1093/ecco-jcc/jjaf100 (PMC12231548; doi:10.1093/ecco-jcc/jjaf100)
Supplement: jjaf100_suppl_Supplementary_Tables_S1-S6_Figure_S1 [file jjaf100_suppl_supplementary_tables_s1-s6_figure_s1.docx]

Table S1. Endpoint descriptions and definitions.

| **Assessment** | **Definitions** |
| --- | --- |
| FACIT-Fatigue | 13 items; range 0-52; higher scores indicate less fatigue; clinically meaningful improvement was identified as a  6- to 9-point or greater increase in the FACIT-Fatigue total score |
| SF | SF: number of liquid or very soft stools defined using the Bristol Stool Scale Category 6 or 7, that is, liquid or watery stools  Endpoint measured as change from baseline in SF at timepoint of interest |
| AP | AP [4-point scale: 0=none, 1=mild, 2=moderate, 3=severe]  Endpoint measured as change from baseline in AP at timepoint of interest |
| Clinical response by PRO | defined as ≥30% decrease in very soft or liquid stool frequency (SF) or abdominal pain (AP) and neither worse than baseline |
| Clinical remission by CDAI | CDAI <150 |
| Endoscopic response | ≥50% reduction from baseline in SES-CD |
| Endoscopic Remission | SES-CD ≤4, and ≥2-point reduction from baseline, and no subscore >1, defined as no subscore >1 in any individual variable |
| Urgency Numeric Rating Scale | Measures the sudden need for bowel movement; range 0-10; higher scores indicate more severe bowel urgency |
| IBDQ | Measures health-related quality of life through symptoms directly related to the primary bowel disturbance, systemic symptoms, emotional function, and social function; range 32-224; higher scores indicate better quality of life  IBDQ response: ≥16 point improvement from baseline in IBDQ score  IBDQ remission: IBDQ score ≥170 |
| QIDS-SR16 | Assesses existence and severity of symptoms of depression; 16 items; range 0-27; higher scores indicate greater symptom severity |
| SF-36 | 36-item, patient-reported survey that measures health-related quality of life, contains Physical and Mental Component Summary (PCS and MCS) scores; range 0-100; higher scores indicate better health |
| C-reactive protein | Endpoint measured as change from baseline at time point of interest |
| Fecal calprotectin | Endpoint measured as change from baseline at time point of interest |
| IBD-DI total score | Measures IBD-related disability; 15 items; range 0-100; higher scores indicate greater levels of IBD-related disability |
| PGRS score | 1-item patient-rated questionnaire designed to assess the patients’ rating of their disease symptom severity over the past 24 hours |
| DSI-CD total score | Instrument that encompasses features of the disease phenotype, inflammatory disease activity (measured by endoscopy, cross-sectional imaging, and biomarkers) and the impact of the disease |
| Abbreviations: FACIT-Fatigue = Functional Assessment of Chronic Illness Therapy-Fatigue; SF = stool frequency; AP = abdominal pain; PRO = Patient Reported Outcome; CDAI = Crohn’s Disease Activity Index; IBDQ = Inflammatory Bowel Disease Questionnaire; QIDS-SR16 = 16-item  quick inventory of depressive symptomatology - self-report; SF-36 = Short Form Survey; IBD-DI = Inflammatory Bowel Disease-Disability Index; PGRS = Patient Global Rating of Severity; DSI-CD = Disease Severity Index-Crohn’s Disease | |

Table S2. Baseline summary of mirikizumab and risankizumab populations. Individual patient-level data from mirikizumab were matched against the aggregate data from risankizumab. After matching, the re-weighted mirikizumab population aligns closely with the target risankizumab population.

| Variable | Risa 600mg+PBO (target) | Miri+PBO, before | Miri+PBO, after | Risa 1200mg+PBO (target) | Miri+PBO, before | Miri+PBO, after |
| --- | --- | --- | --- | --- | --- | --- |
| N/ESS | 889 | 764 | 323 | 892 | 764 | 330 |
| Age, mean (SD) | 38.7 (13.4) | 36.2 (13.1) | 38.7 (13.4) | 38.0 (13.3) | 36.2 (13.1) | 38.0 (13.3) |
| Male, prop | 0.526 | 0.58 | 0.526 | 0.529 | 0.58 | 0.529 |
| White, prop | 0.821 | 0.723 | 0.821 | 0.797 | 0.723 | 0.797 |
| Disease duration, years, mean (SD) | 10.0 (8.6) | 7.4 (8.0) | 10.0 (8.6) | 10.1 (8.8) | 7.4 (8.0) | 10.1 (8.8) |
| CDAI, mean (SD) | 314.4 (63.7) | 322.3 (85.0) | 314.4 (63.8) | 314.9 (65.5) | 322.3 (85.0) | 314.9 (65.6) |
| Corticosteroid use, prop | 0.321 | 0.297 | 0.321 | 0.315 | 0.297 | 0.315 |
| Prior biologic fail, prop | 0.393 | 0.479 | 0.393 | 0.402 | 0.479 | 0.402 |
| FACIT-Fatigue, mean (SD) | 23.8 (11.0) | 31.7 (11.5) | 23.8 (11.0) | 24.3 (11.2) | 31.7 (11.5) | 24.3 (11.2) |
| IBDQ, mean (SD) | 119.2 (31.2) | 128.7 (33.0) | 119.2 (31.2) | 121.4 (32.3) | 128.7 (33.0) | 121.4 (32.3) |
| Disease location, prop |  |  |  |  |  |  |
| Ileal only | 0.146 | 0.11 | 0.146 | 0.135 | 0.11 | 0.135 |
| Colonic only | 0.375 | 0.384 | 0.375 | 0.376 | 0.384 | 0.376 |
| Immunomodulator use, prop | 0.232 | 0.263 | 0.232 | 0.233 | 0.263 | 0.233 |
| *CRP <8.6, prop | 0.5 | 0.517 | 0.5 | 0.5 | 0.541 | 0.5 |
| *FC- <1128.6, prop | 0.5 | 0.365 | 0.5 | 0.5 | 0.363 | 0.5 |
| Abbreviations: Miri = mirikizumab; Risa = risankizumab; PBO = placebo; CD = Crohn’s Disease; FACIT-F = Functional Assessment of Chronic Illness Therapy-Fatigue; CRP = C-reactive protein; FCP = Fecal calprotectin; prop = proportion; SD = standard deviation; ESS = Effective Sample Size; IBDQ = Inflammatory Bowel Disease Questionnaire; CDAI= Crohn’s Disease Activity Index.  *For Risa 1200mg+PBO, CRP <9.28 and FCP<1113.1 | | | | | | |

Table S3. Anchored MAIC of induction efficacy between mirikizumab and risankizumab

| Mirikizumab vs Risankizumab (600 mg) at Week 12 | | | | | | | |
| --- | --- | --- | --- | --- | --- | --- | --- |
| Method | Target (PBO, N=362, response rate) | Target (Risa, N=527*, response rate) | VIVID-1 (PBO, N=193, response rate) | VIVID-1 (Miri, N=571, response rate) | Odds Ratio (95% CI) | SE (log (OR)) | p-value |
| Unadjusted | 0.345 | 0.486 | 0.223 | 0.324 | 0.933 (0.583 to 1.495) | 0.240 | 0.774 |
| MAIC | 0.345 | 0.486 | 0.323  (ESS=61) | 0.473  (ESS=262) | 1.037 (0.483 to 2.227) | 0.390 | 0.925 |
| Mirikizumab vs Risankizumab (1200 mg) at Week 12 | | | | | | | |
| Unadjusted | 0.345 | 0.485 | 0.223 | 0.324 | 0.937 (0.585 to 1.500) | 0.240 | 0.785 |
| MAIC | 0.345 | 0.485 | 0.315  (ESS=66) | 0.462  (ESS=264) | 1.060 (0.501 to 2.243) | 0.382 | 0.879 |
| OR = odds ratio; CI = confidence interval; SE = standard error, MAIC = matching-adjusted indirect comparison; ESS = Effective Sample Size; Miri = mirikizumab; Risa = risankizumab; PBO = placebo.  Individual patient data (IPD) from the VIVID-1 trial of Miri were weighted to match the aggregate data for the target trials of Risa. Relevant baseline effect modifiers for adjustment included age, male, white race, CD duration (years), CDAI total score at baseline, corticosteroid use, prior biologic failure, FACIT-F score at baseline, IBDQ total score at baseline, disease location, immunomodulator use, CRP, and fecal calprotectin. MAIC estimator incorporating the weights for IPD was used to compare the outcome between Miri and Risa using a weighted logistic regression model. An unadjusted indirect comparison estimator, which directly compared treatment groups without adjustments for differences in trial populations, was also included for comparison using Bucher’s method.  Robust sandwich estimator was used for SE in MAIC analysis. After-matching ESS was shown in MAIC row, and (before-matching) sample size was indicated by N.  *N=530 for Mirikizumab vs Risankizumab (1200 mg) not 527 | | | | | | | |

Table S4: Pearson correlation coefficients of baseline variables versus fatigue

|  | Importance | Correlation | |
| --- | --- | --- | --- |
| IBDQ systemic symptoms | 47.36 | 0.784 | |
| QIDS-SR16 | 16.52 | -0.689 | |
| SF-36 MCS | 12.37 | 0.654 | |
| IBDQ emotional function | 9.87 | 0.686 | |
| SF-36 PCS | 9.45 | 0.579 | |
| IBD-DI total score | 4.20 | -0.566 | |
| IBDQ social function | 3.24 | 0.603 | |
| IBDQ bowel symptoms | 1.35 | 0.538 | |
| PGRS score | 0.42 | -0.306 | |
| CDAI total score | 0.41 | -0.285 | |
| Fecal calprotectin | 0.35 | -0.051 | |
| Abdominal pain score | 0.32 | -0.260 | |
| Bowel urgency NRS | 0.32 | -0.135 | |
| DSI-CD total score | 0.27 | -0.021 | |
| SES-CD total score | 0.19 | 0.024 | |
| C-reactive protein | 0.16 | -0.080 | |
| Age at Crohn’s Disease Diagnosis (years) | 0.14 | -0.070 | |
| Stool frequency score | 0.07 | -0.125 | |
| Hematocrit (%) | 0.05 | 0.086 | |
| Number of surgical bowel resection | 0.05 | 0.069 | |
| Baseline corticosteroid use | 0.03 | NA | |
| Baseline immunomodulator use | -0.01 | NA | |
| Hemoglobin (g/dL) | -0.03 | 0.081 | |
| Duration of CD (<1 year; 1 to 5 years; ≥ 5 years) | -0.06 | NA | |
| Disease location (ileal, colonic, ileal-colonic) | -0.06 | NA | |
| Variable Importance was measured by the percentage increase in mean squared error after permuting a specific predictor in the random forest; higher percentages indicate greater importance. Absolute value of Pearson correlation 0.10-0.30 as weak, >0.30-0.50 as moderate, and >0.50 to 1 as strong. NA denotes that Pearson correlation is not available for categorical variables.  Abbreviations: Miri = mirikizumab; IBDQ = Inflammatory Bowel Disease Questionnaire; IBD-DI = Inflammatory Bowel Disease-Disability Index; PGRS = Patient Global Rating of Severity; NRS = numeric rating scale; DSI-CD = Disease Severity Index-Crohn’s Disease; SES-CD= Simple Endoscopic Score for Crohn’s Disease; CDAI = Crohn’s Disease Activity Index; CRP = C-reactive protein | | |  |

Table S5. Pearson Correlation Coefficients of change from baseline for clinical outcomes versus FACIT-Fatigue change from baseline at Weeks 12 and 52.

|  | Pearson Correlation | 95% Confidence Interval |
| --- | --- | --- |
| Week 12 | | |
| Abdominal Pain Average Score | -0.351 | -0.421 to -0.278 |
| Stool Frequency Average Score | -0.267 | -0.341 to -0.189 |
| Urgency NRS | -0.287 | -0.360 to -0.210 |
| Fecal Calprotectin (log-transformed) | -0.026 | -0.117 to 0.065 |
| CRP (log transformed) | -0.161 | -0.240 to -0.080 |
| IBDQ total score | 0.652 | 0.603 to 0.697 |
| IBDQ bowel symptoms score | 0.515 | 0.452 to 0.573 |
| IBDQ systemic symptoms score | 0.702 | 0.659 to 0.742 |
| IBDQ emotional function score | 0.613 | 0.559 to 0.661 |
| IBDQ social function score | 0.570 | 0.512 to 0.622 |
| SF-36 PSC score | 0.473 | 0.407 to 0.534 |
| SF-36 MSC score | 0.580 | 0.523 to 0.632 |
| QIDS score | -0.572 | -0.625 to -0.513 |
| CDAI total score | -0.386 | -0.454 to -0.314 |
| SES-CD total score | -0.149 | -0.228 to -0.069 |
| HCT (%) | 0.002 | -0.080 to 0.083 |
| Hb (g/dL) | 0.024 | -0.058 to 0.106 |
| RHI | -0.163 | -0.242 to -0.082 |
| Week 52 | | |
| Abdominal Pain Average Score | -0.379 | -0.447 to -0.307 |
| Stool Frequency Average Score | -0.306 | -0.379 to -0.230 |
| Urgency NRS | -0.361 | -0.430 to -0.288 |
| Fecal Calprotectin (log-transformed) | -0.112 | -0.201 to -0.020 |
| CRP (log transformed) | -0.208 | -0.285 to -0.128 |
| IBDQ total score | 0.716 | 0.673 to 0.753 |
| IBDQ bowel symptoms score | 0.574 | 0.517 to 0.627 |
| IBDQ systemic symptoms score | 0.738 | 0.699 to 0.773 |
| IBDQ emotional function score | 0.703 | 0.659 to 0.742 |
| IBDQ social function score | 0.635 | 0.583 to 0.681 |
| SF-36 PSC score | 0.574 | 0.517 to 0.627 |
| SF-36 MSC score | 0.631 | 0.579 to 0.678 |
| QIDS score | -0.606 | -0.660 to -0.545 |
| CDAI total score | -0.446 | -0.509 to -0.378 |
| SES-CD total score | -0.196 | -0.273 to -0.116 |
| HCT (%) | 0.115 | 0.033 to 0.194 |
| Hb (g/dL) | 0.114 | 0.033 to 0.194 |
| RHI | -0.171 | -0.250 to -0.090 |
| N=579; all clinical outcomes used mBOCF except QIDS score which was observed values.  Abbreviations: N = number of patients in the analysis population; mBOCF = modified baseline observation carried forward; NRS = numeric rating scale; SES-CD = Simple Endoscopic Score for Crohn's Disease; CDAI = Crohn's Disease Activity Index; CRP = C-reactive protein; IBDQ = Inflammatory Bowel Disease Questionnaire; PCS = physical component summary score;  MCS = mental component summary score; QIDS = Quick Inventory of Depressive Symptomatology; HCT = Hematocrit; Hb = Hemoglobin;  RHI = Robarts Histopathology Index(Total Colon). | | |

Table S6. IBDQ domain categories

| Domain | Medical Constructs Included |
| --- | --- |
| Bowel Symptoms | BM frequency, Loose BMs, Abdominal cramps, Abdominal pain, Flatulence, Bloating, Rectal bleeding, Needing the toilet with empty bowels, Accidental soiling, Nausea |
| Systemic Symptoms | Fatigue, Energy level, Generally unwell, Trouble sleeping, Weight loss/maintenance |
| Emotional Function | Frustration/impatience/restlessness, Worries about surgery, Fear of not finding a bathroom, Depression, Anxiety, Relaxation, Embarrassment, Tearful/upset, Anger, Irritability, Lack of understanding from others, Satisfaction of personal life |
| Social Function | Unable to attend school/work, Delayed or cancelled social engagement, Difficulty playing sports, Avoiding events with no bathroom, Sexual activity |
| Abbreviations: BM=bowel movement  Adapted from Guyatt et al. ^1^ | |


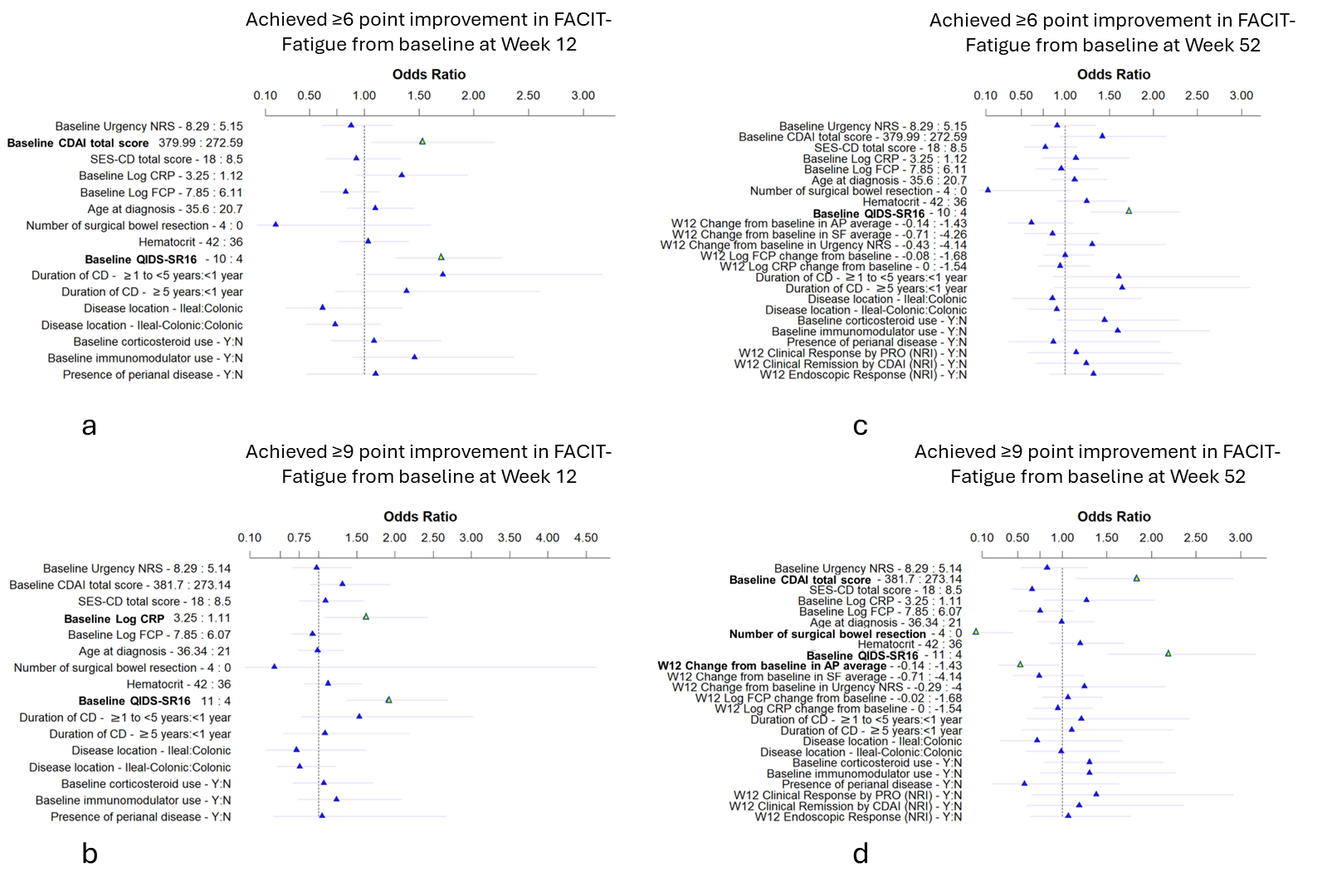
Figure S1. Summary of odds ratios (OR) and confidence intervals (CI) for achieving FACIT-Fatigue improvement of ≥6 or ≥9 at week 12 or 52 from multivariable logistic regression. Interquartile-range odds ratios for continuous predictors and simple odds ratios for categorical predictors. Numbers at left are upper quartile: lower quartile for continuous variables or current group: reference group for categorial variables. The bars represent 95% confidence intervals.

Predictors in bold with highlighted triangles are significant.

References

1. Guyatt G, Mitchell A, Irvine EJ, Singer J, Williams N, Goodacre R, Tompkins C. A new measure of health status for clinical trials in inflammatory bowel disease. *Gastroenterology* 1989; **96**(3): 804-10.
